# Supplementary material for: Signatures of mitonuclear coevolution in a warbler species complex
Source: Nat Commun. 2021 Jul 13;12:4279. doi: 10.1038/s41467-021-24586-8 (PMC8277850; doi:10.1038/s41467-021-24586-8)
Supplement: Supplementary file 1 — Supplementary Information [file 41467_2021_24586_MOESM1_ESM.docx]

**SUPPLEMENTARY MATERIAL**

**Signature of selective sweep**

The bootstrap mean of π within the candidate gene block was significantly lower than the rest of the chr5 in Haida Gwaii (Fig. S4 AD, 95% CI of mean π, candidate block = 0.0012-0.0022, versus rest chr5 = 0.0037-0.0039), Valdez (Fig. S4 BE, 95% CI of mean π, candidate block = 0.0012-0.0020, versus rest chr5 = 0.0035-0.0037), and other coastal STOW (Fig. S4 CF, 95% CI of mean π, candidate block = 0.0013-0.0022, versus rest chr5 = 0.0034-0.0036). We focus on the region 2.5Mb and 7Mb within chr5 to acquire high resolution LD landscape around the candidate genetic region (between ~ 3.73-5.17 Mb). LD was significantly higher within the candidate gene block than its surrounding genetic region (between 2.5-7Mb) on chr5 within Haida Gwaii (Fig. S4 G, 95% CI of mean r^2^, candidate block 0.1567-0.1756, versus rest = 0.1287-0.1301), Valdez (Fig. S4 H, 95% CI of mean r^2^, candidate block = 0.1801-0.1982, versus rest = 0.0898-0.0907), as well as the other coastal STOW (Fig. S4 I, 95% CI of mean r^2^, candidate block = 0.0990-0.1093, versus rest = 0.0430-0.0434).

**Testing any potential sex linkage of the chr5 candidate gene block**

For nuclear genomic regions that are sex linked but not directly functionally associated with mitochondrial functions, they could be correlated with mitochondrial ancestry. To test this potential artifact, we tested whether the ancestry of the chr5 candidate block is associated with sex. In particular, we ran independent t-test of chr5 candidate gene block ancestry between females and males. There was no significant sex effect in the candidate gene block (t = -0.27, *p* = 0.7923). Therefore, there is no evidence that the mitonuclear association discovered in this study is due to sex linkage of the chr5 gene block. The directional functional association of genes within this chr5 gene block (involved in ND2 and ATP related biochemistry that matches with mtDNA functional differentiation, Table S1-S3) and signatures of selective sweep within this chr5 gene block further suggest that the mitonuclear association we have characterized reflects mitonuclear coadaptation.


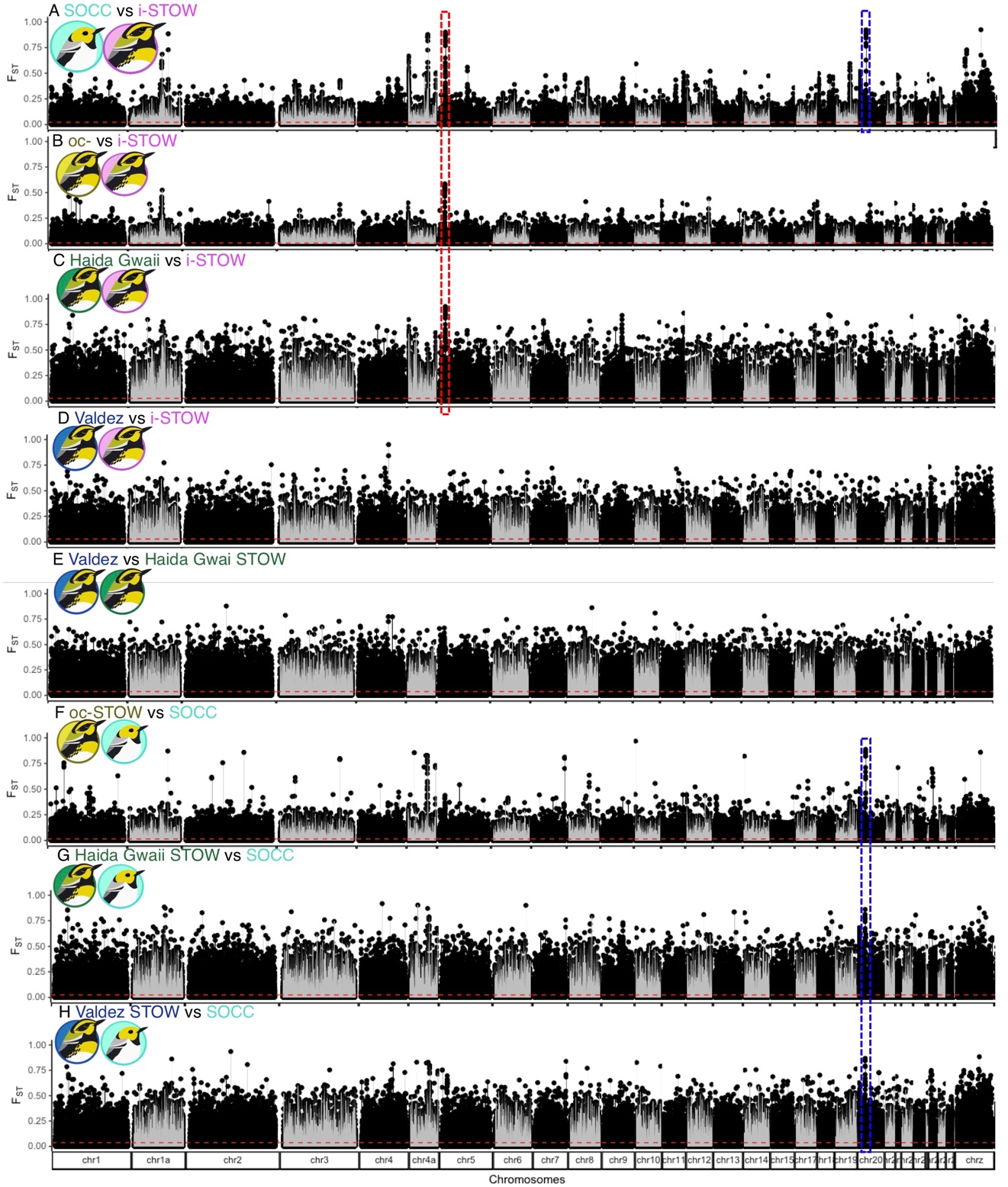


**Fig. S1** Genetic differentiation (*F_ST_*) across the genome, between pairs of *S. occidentalis* (SOCC) and/or *S. townsendi* (STOW) populations. A distinctive differentiation peak was found between coastal STOW (Haida Gwaii and oc-STOW) and inland STOW that reside in chromosome 5 (red boxes, **A**-**C**). The ASIP-RALY peak demonstrates consistent differentiation between SOCC and various STOW (blue boxes, A, **F**-**H**). Red horizontal dashed lines reflect Weir & Cockerham weighted average *F_ST_*.


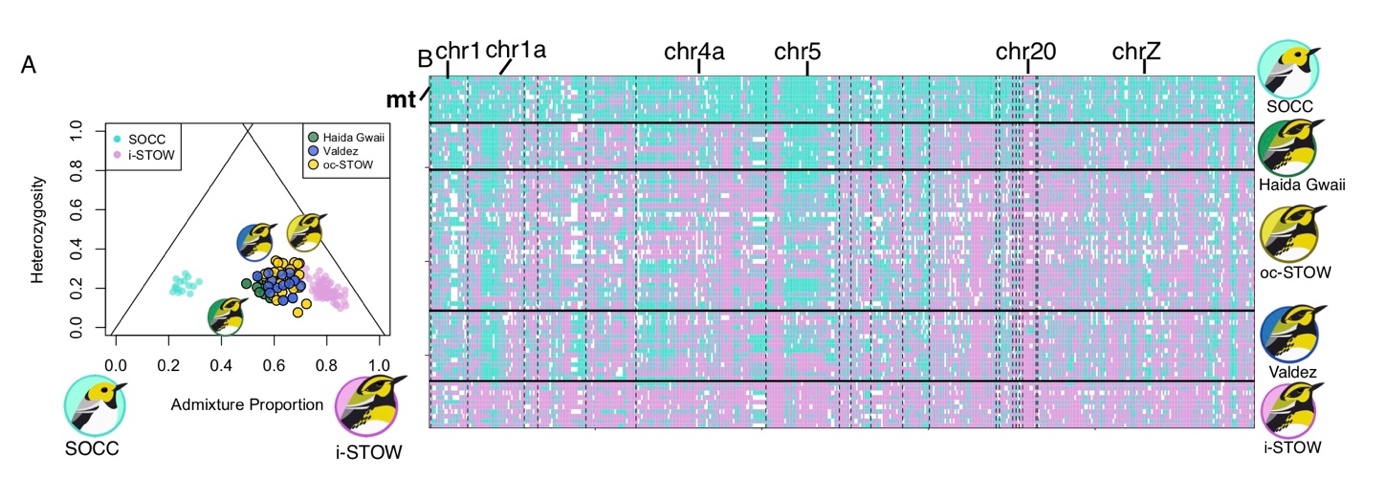
**Fig. S2** Admixture signature of coastal STOW populations. **(A)** Triangle plot showing relationship between individual admixture proportion vs. heterozygosity based on 618 SNPs (*F_ST_* > 0.3) between SOCC and inland STOW, showing that coastal STOW populations consistent with admixtures between SOCC and inland STOW-like population. **(B)** ancestry score (homozygotes SOCC and inland STOW respectively in turquoise and magenta, heterozygotes in light blue) of mtDNA and 618 nDNA SNPs (with *F_ST_* > 0.3) in randomly sampled 10 individuals from parental populations (SOCC, inland STOW), and different coastal STOW populations.

**Fig. S3** The Weir & Cockerham weighted *F_ST_* among SOCC, inland and coastal STOW. Each double-head arrow represents a pairwise comparison among the populations. The populations are oriented according to their relative geographical locations. OC-STOW corresponds to other coastal STOW that is not from Valdez or Haida Gwaii. The widths of the arrows are weighted by the *F_ST_* between each pair of populations. Surprisingly some coastal STOW populations demonstrate greater differentiation from the parental populations than between the parental populations (*F_ST_* = 0.0226), which could be due to greater drift in small population size.

**Fig. S4 Signature of selective sweep within the mtDNA-associated chr5 gene block in each of the coastal STOW population. A-C** bootstrap distribution of mean π within candidate gene block (coral) versus the rest of chr5 (green, blue, and yellow for different coastal STOW populations). **D-E** window-based π cross the 2.5-7Mb block around the candidate gene block (red boxes) on chr5 in each of the coastal STOW population. **G-I** pairwise LD (r^2^) within the 2.5-7Mb block outside the candidate gene block (coral triangles) on chr5 in each of the coastal STOW population.

**
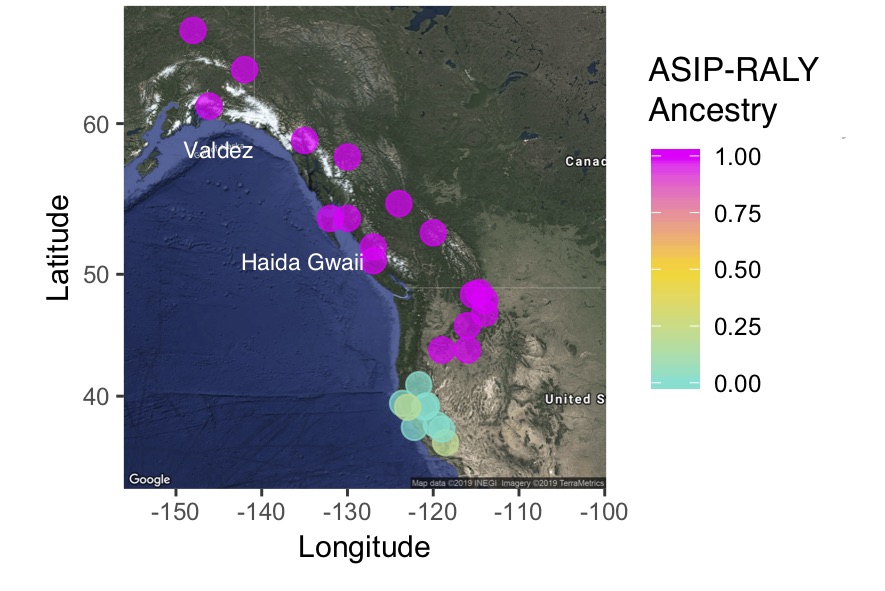
**

**Fig. S5** Geographical distribution of ASIP-RALY ancestry. Map data ©2019 Google.

**
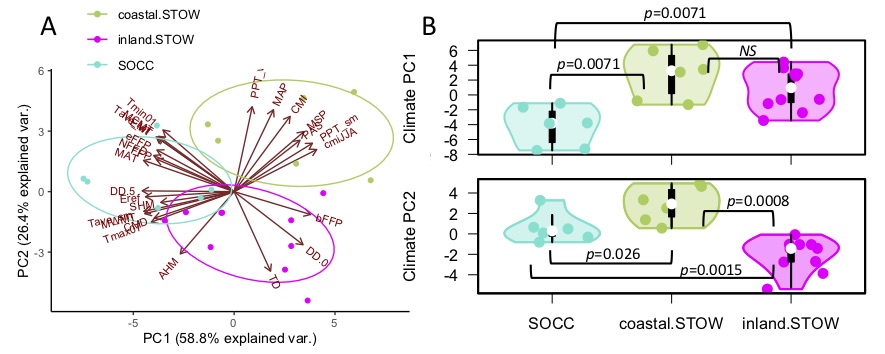
Fig. S6 Dissecting climate PCA**: **A**, biplot of PCA demonstrating loadings of the 5 climate variables in the PC space. **B**, Violin plots showing distribution of climate PC1 (top) and PC2 (bottom) among sampling sites. There was significant difference in climate PC1 and PC2 among SOCC (turquoise), coastal (green) and inland STOW (magenta) habitats (Kruskal-Wallis test followed by pairwise Wilcoxon rank sum test with Benjamini-Hochberg multiple hypothesis correction, *p* < 0.05). Corrected *p* values of each pairwise comparison is listed between distributions in the violine plots. The lower and upper bounds of violin plots show are respectively the 1^st^ and 3^rd^ quartiles around medians.

**Table S1** Divergence of mitochondrial genes between SOCC and inland STOW.

| **Name** | **Start** | **Stop** | **Length** | **%Fixed difference** | **Number of Amino Acid Changes** |
| --- | --- | --- | --- | --- | --- |
| ND1 | 2829 | 3776 | 948 | 0.738 | 0 |
| ND2 | 4009 | 5043 | 1035 | 0.966 | 4 |
| COX1 | 5417 | 6949 | 1533 | 0.457 | 0 |
| COX2 | 7105 | 7779 | 675 | 0.741 | 0 |
| ATP8 | 7861 | 8022 | 162 | 0.617 | 0 |
| ATP6 | 8019 | 8699 | 681 | 0.441 | 1 |
| COX3 | 8708 | 9490 | 783 | 0.639 | 0 |
| ND3 | 9561 | 9908 | 348 | 1.149 | 0 |
| ND4L | 9984 | 10277 | 294 | 0.340 | 0 |
| ND4 | 10274 | 11641 | 1368 | 0.439 | 0 |
| ND5 | 11870 | 13660 | 1791 | 0.782 | 0 |
| COB | 13684 | 14817 | 1134 | 0.617 | 0 |
| ND6 | 14996 | 15511 | 516 | 1.357 | 0 |

**Table S2** Analysis of five mitochondrial substitutions separating the SOCC and inland STOW haplotypes. Potential impacts of each substitution are assessed using PROVEAN (both forwards and reverse mutations), TreeSAAP, and MEME. Analyses with evidence for positive selection are highlighted in bold.

| Gene | Position | STOW allele | SOCC allele | *S. coronata*   allele | Mutation | PROVEAN score | Impact | Reverse Mutation | PROVEAN score | Impact | TreeSAAP Category | MEME *p*-value |
| --- | --- | --- | --- | --- | --- | --- | --- | --- | --- | --- | --- | --- |
| ND2 | 11 | T | A | A | A11T | 0.691 | Neutral | T11A | -0.699 | Neutral | **-** | 0.67 |
| ND2 | 150 | N | D | N | **N150D** | **-3.964** | **Non-neutral** | D150N | 3.605 | Neutral | **Alpha-helical Tendencies** | 0.67 |
| ND2 | 320 | P | S | S | S320P | 2.597 | Neutral | **P320S** | **-2.888** | **Non-neutral** | **-** | 0.67 |
| ND2 | 324 | A | T | T | T324A | -0.396 | Neutral | A324T | 0.393 | Neutral | **-** | 0.41 |
| ATP6 | 25 | T | M | T | T25M | 0.178 | Neutral | M25T | 0.246 | Neutral | **-** | **< 0.0001** |

**Table S3** Genomic cline parameter *α* and *β* estimates for testing directional and divergent selection of chr5 candidate gene block. P-values (Bonferroni correction for multiple hypotheses testing) represent two-sided T tests against the null hypotheses that *α* or *β* is zero.

|  | *α* | **SE** (*α* ) | ***p-value*** | *β* | **SE** ( *β* ) | ***p-value*** |
| --- | --- | --- | --- | --- | --- | --- |
| **mtDNA** | -0.3388 | 0.1002 | 0.0036 | 0.7662 | 0.246 | 0.0008 |
| **Chr5 candidate** | -0.2766 | 0.0395 | < 10^-10^ | 0.7694 | 0.0983 | < 10^-12^ |

**Table S4** Definitions of 26 climate variables from ClimateWNA^1^. The loading of variables in the climate PCA sorted by their loading along principal components (PC1-5). The signs represent whether the variable is positively (+) or negatively (-) associated with each climatic PC.

| **Variable abbreviation** | **Variable Name** | **PC1** | **PC2** | **PC3** | **PC4** | **PC5** |
| --- | --- | --- | --- | --- | --- | --- |
| AHM | annual heat-moisture index | -0.14 | -0.25 | -0.12 | 0.08 | 0.50 |
| bFFP | The day of the year on which FFP begins | 0.20 | -0.09 | -0.39 | 0.20 | -0.12 |
| CMD | Climatic moisture deficit | -0.21 | -0.11 | -0.35 | -0.09 | -0.14 |
| CMI | Climatic moisture index | 0.15 | 0.30 | -0.08 | -0.16 | 0.07 |
| cmiJJA | Hogg's summer (June-Aug) climate moisture index | 0.22 | 0.17 | 0.18 | -0.05 | 0.05 |
| DD.0 | degree-days below 0°C, chilling degree-days | 0.18 | -0.21 | 0.15 | -0.33 | -0.13 |
| DD.5 | degree-days above 5°C, growing degree-days | -0.24 | 0.00 | -0.01 | -0.26 | 0.05 |
| eFFP | The day of the year on which FFP begins | -0.20 | 0.16 | 0.29 | -0.14 | -0.08 |
| EMT | Extreme minimum temperature over 30 years | -0.21 | 0.22 | 0.07 | 0.06 | 0.01 |
| Eref | Hargreaves reference evaporation (mm) | -0.23 | -0.02 | -0.28 | -0.04 | -0.05 |
| FFP | Frost-free period | -0.21 | 0.13 | 0.34 | -0.17 | 0.03 |
| MAP | Mean annual precipitation (mm) | 0.10 | 0.32 | -0.16 | -0.21 | 0.09 |
| MAT | Mean annual temperature | -0.24 | 0.12 | -0.06 | 0.05 | 0.09 |
| MCMT | Mean coldest month temperature (°C) | -0.20 | 0.22 | -0.08 | 0.19 | -0.03 |
| MSP | May through September precipitation | 0.19 | 0.24 | -0.06 | -0.15 | 0.28 |
| MWMT | Mean winter temperature | -0.24 | -0.09 | -0.06 | -0.22 | 0.21 |
| NFFD | number of frost-free days | -0.21 | 0.14 | 0.30 | -0.08 | 0.02 |
| PAS | precipitation as snow | 0.17 | 0.20 | -0.30 | -0.24 | 0.06 |
| PPT_sm | summer precipitation (mm) | 0.21 | 0.19 | -0.02 | -0.14 | 0.26 |
| PPT_wt | winter precipitation (mm) | 0.05 | 0.34 | -0.21 | -0.23 | -0.10 |
| SHM | summer heat-moisture index ((MWMT)/(MSP/1000)) | -0.19 | -0.04 | -0.14 | -0.37 | -0.57 |
| Tave_sm | summer mean temperature (°C) | -0.24 | -0.08 | -0.05 | -0.19 | 0.23 |
| Tave_wt | winter mean temperature (°C) | -0.20 | 0.21 | -0.07 | 0.19 | -0.01 |
| TD | Continentality | 0.10 | -0.31 | 0.07 | -0.36 | 0.16 |
| Tmax07 | winter mean maximum temperature (°C) | -0.22 | -0.12 | -0.25 | -0.13 | 0.24 |
| Tmin01 | winter mean minimum temperature (°C) | -0.19 | 0.24 | -0.04 | 0.19 | 0.04 |

**Table S5**

| Common adaptor | plus strand | 5’-ACACTCTTTCCCTACACGACGCTCTTCCGATCTxxxx TGCA-3’ |
| --- | --- | --- |
|  | minus strand | 5’-xxxxAGATCGGAAGAGCGTCGTGTAGGGAAAGAGTGT-3’ |
| Barcode adaptor | plus strand | 5’-AGATCGGAAGAGCGGTTCAGCAGGAATGCCGAG-3’ |
|  | minus strand | 5’-CTCGGCATTCCTGCTGAACCGCTCTTCCGATCTTGCA-3’ |

**Supplementary data1** Names and functions of genes involved in the 1.2 Mb chromosome 5 island of differentiation between inland STOW and coastal STOWs, as well as between inland STOW and SOCC. The Gene Ontology molecular and biological functions were based on *Taeniopygia guttata* homolog ^2^ from UniProt ^3^.

(Attachment)

**Supplementary data 2** Tab1: Accession numbers for *ND2* sequences used to evaluate evidence for positive selection with MEME. Tab2: Accession numbers for *ATP6* sequences used to evaluate evidence for positive selection with MEME.

(Attachment)

**Supplementary data 3** TreeSAAP analyses of physicochemical property of *ND2* and *ATP6*.

(Attachment)

**Reference**

1. Wang, T., Hamann, A., Spittlehouse, D. L. & Murdock, T. Q. ClimateWNA-high-resolution spatial climate data for western North America. *J. Appl. Meteorol. Climatol.* **51**, 16–29 (2012).

2. Johnson, M. *et al.* NCBI BLAST: a better web interface. *Nucleic Acids Res.* **36**, W5–W9 (2008).

3. Bateman, A. UniProt: A worldwide hub of protein knowledge. *Nucleic Acids Res.* **47**, D506-515 (2019).
