# Supplementary material for: Signatures of mitonuclear coevolution in a warbler species complex
Source: Nat Commun. 2021 Jul 13;12:4279. doi: 10.1038/s41467-021-24586-8 (PMC8277850; doi:10.1038/s41467-021-24586-8)
Supplement: Supplementary file 3 — Reporting Summary [file 41467_2021_24586_MOESM3_ESM.pdf]

nature research

Corresponding author(s): Silu Wang, NCOMMS-21-05866

Last updated by author(s): 2021/02/18

Reporting Summary

Nature Research wishes to improve the reproducibility of the work that we publish. This form provides structure for consistency and transparency in reporting. For further information on Nature Research policies, see our Editorial Policies and the Editorial Policy Checklist.

Statistics

For all statistical analyses, confirm that the following items are present in the figure legend, table legend, main text, or Methods section.

n/a	Confirmed	
☐	☒	The exact sample size (*n*) for each experimental group/condition, given as a discrete number and unit of measurement
☐	☒	A statement on whether measurements were taken from distinct samples or whether the same sample was measured repeatedly
☐	☒	The statistical test(s) used AND whether they are one- or two-sided<br>*Only common tests should be described solely by name; describe more complex techniques in the Methods section.*
☐	☒	A description of all covariates tested
☐	☒	A description of any assumptions or corrections, such as tests of normality and adjustment for multiple comparisons
☐	☒	A full description of the statistical parameters including central tendency (e.g. means) or other basic estimates (e.g. regression coefficient) AND variation (e.g. standard deviation) or associated estimates of uncertainty (e.g. confidence intervals)
☐	☒	For null hypothesis testing, the test statistic (e.g. *F*, *t*, *r*) with confidence intervals, effect sizes, degrees of freedom and *P* value noted<br>*Give P values as exact values whenever suitable.*
☒	☐	For Bayesian analysis, information on the choice of priors and Markov chain Monte Carlo settings
☒	☐	For hierarchical and complex designs, identification of the appropriate level for tests and full reporting of outcomes
☐	☒	Estimates of effect sizes (e.g. Cohen's *d*, Pearson's *r*), indicating how they were calculated

Our web collection on statistics for biologists contains articles on many of the points above.

Software and code

Policy information about availability of computer code

Data collection	No software was used to collect data in this study.
Data analysis	To process genotyping-by-sequencing (GBS) data, we trimmed the data with Trimmomatic, aligned the reads with bwa, did SNP-calling with GATK, filtered SNPs and calculated Fst with VCFtools. PCA analysis was done with SNPRelate in R, and mt-GWAS was done with GenABEL in R. Gene function analysis was done in UniProt. mtDNA sequence analysis was done in samtools, bcftools, seqtk, and Geneious. Partial mantel test was done in R.

For manuscripts utilizing custom algorithms or software that are central to the research but not yet described in published literature, software must be made available to editors and reviewers. We strongly encourage code deposition in a community repository (e.g. GitHub). See the Nature Research guidelines for submitting code & software for further information.

Data

Policy information about availability of data

All manuscripts must include a data availability statement. This statement should provide the following information, where applicable:

- Accession codes, unique identifiers, or web links for publicly available datasets
- A list of figures that have associated raw data
- A description of any restrictions on data availability

The sequence data generated in this study have been deposited in the GenBank SRA database under accession codes PRJNA573930 [https://www.ncbi.nlm.nih.gov/bioproject/PRJNA573930/] and PRJNA642412 [https://www.ncbi.nlm.nih.gov/bioproject/PRJNA642412/]. The processed genomic data are available at Dryad (https://doi.org/10.5061/dryad.44j0zpc9t).

Field-specific reporting

Please select the one below that is the best fit for your research. If you are not sure, read the appropriate sections before making your selection.

☐ Life sciences ☐ Behavioural & social sciences ☒ Ecological, evolutionary & environmental sciences

For a reference copy of the document with all sections, see nature.com/documents/nr-reporting-summary-flat.pdf

Ecological, evolutionary & environmental sciences study design

All studies must disclose on these points even when the disclosure is negative.

Study description	The core of this study is mitonuclear coevolution in ancient hybrid populations. We sampled N=47 and N=81 individuals from either of the parental populations and N=95 individuals from the ancient hybrid population. We found population structure within the hybrid population, and thus corrected for substructure with a kinship matrix when testing association between mtDNA and nDNA. We have also tested the association of mtDNA and nDNA association across sampling sites, and corrected for spatial autocorrection with a partial mantel test. Sampling is hierarchically nested, where >5 individuals were sampled from each sampling site which compose three sampling regions, the hybrid region and two parental regions.
Research sample	We sampled N=47 Setophaga occidentalis, N= 81 Setophaga townsendi, N = 95 ancient hybrids of the two. All individuals were breeding adults (which is important for migratory birds). Details on sex, locations, genotype information is summarized in SOCC.STOW.197.genomicEV.mt.sex.csv in Dryad: https://doi.org/10.5061/dryad.44j0zpc9t<br>Most of the ancient hybrids and inland townsendi were from published sampling (Krosby & Rohwer 2019).<br>Krosby, M. & Rohwer, S. A 2000 km genetic wake yields evidence for northern glacial refugia and hybrid zone movement in a pair of songbirds. Proc. R. Soc. B Biol. Sci. 276, 615–621 (2009).
Sampling strategy	Natural ancient hybrid populations harbor extensive genetic variants and recombinant which allow us to readily identify mitonuclear associations. To ensure sampling representation of natural variation across the range of S. occidentalis and coastal and inland S. townsendi, we sampled sites with even spatial intervals across the ranges. The random sampling was a balanced between within-site replications with individuals (>5) and within-region replications of sampling sites.
Data collection	A set of the samples involved in sequencing was from Burke Museum, the other set (N=54) of the samples were sampled from the field. The museum samples were whole specimens collected by Mead Krosby and Sievert Rohwer (Krosby & Rohwer 2009) and the field blood sample collection was led by Silu Wang in 2015-16 with mist netting (Wang et al. 2019).<br><br>Krosby, M. & Rohwer, S. A 2000 km genetic wake yields evidence for northern glacial refugia and hybrid zone movement in a pair of songbirds. Proc. R. Soc. B Biol. Sci. 276, 615–621 (2009).<br>Wang, S., Rohwer, S., Delmore, K. E. & Irwin, D. E. Cross-decades stability of an avian hybrid zone. J. Evol. Biol. 32, 1242–1251. (2019).
Timing and spatial scale	The field collection was between May 10th and July 15th in 2015-16 in the Pacific West. Sampling took place during the breeding season of the warblers. During breeding season, sampling was conducted continuously in sunny days, as mist netting is non-ethical during rainy days when the warblers are under stress.
Data exclusions	No data were excluded.
Reproducibility	With greater coverage and depth, this study validated the published result from Krosby & Rohwer (2009) that the divergence of mitochondrial DNA between the parental populations was estimated at around 0.5 mya. In addition, the newly generated nuclear genomic data confirms hybrid origin of the coastal S. townsendi populations.
Randomization	This is fundamentally an observational study of geographic variation and the association between genes and phenotypes. Birds cannot be assigned randomly to locations on such a scale. Covariation due to spatial structure was taken into account through consideration of kinship matrices.
Blinding	The sequencing instrument is blind to the identity of each sample.

Did the study involve field work? ☒ Yes ☐ No

Field work, collection and transport

Field conditions	We carried out field work during the breeding seasons of the study species (early May to mid July). The sampling sites were closed coniferous forest where the warblers were active in the canopy, at around 150 feet off the ground. Sampling was not permitted during rainy days.
Location	The collection was carried out in the breeding habitat of the study species.
Access & import/export	We acquired scientific collection permit from U. S. Geological Survey; Departments of Fish & Wildlife of Washington, Idaho, Montana for 2015. Collection permit for California was for 2016. In addition, we acquired importation permit for shipping samples from U.S.A to Canada from Environment Canada.
Disturbance	We tried to minimize the time between capturing and releasing the birds.

Reporting for specific materials, systems and methods

We require information from authors about some types of materials, experimental systems and methods used in many studies. Here, indicate whether each material, system or method listed is relevant to your study. If you are not sure if a list item applies to your research, read the appropriate section before selecting a response.

Materials & experimental systems

n/a	Involved in the study
☒	☐ Antibodies
☒	☐ Eukaryotic cell lines
☒	☐ Palaeontology and archaeology
☐	☒ Animals and other organisms
☒	☐ Human research participants
☒	☐ Clinical data
☒	☐ Dual use research of concern

Methods

n/a	Involved in the study
☒	☐ ChIP-seq
☒	☐ Flow cytometry
☒	☐ MRI-based neuroimaging

Animals and other organisms

Policy information about studies involving animals; ARRIVE guidelines recommended for reporting animal research

Laboratory animals	No lab animals were used in this study.
Wild animals	Birds were lured with song playback, caught with a mist net, and processed within 10 minutes after capture. Briefly, a drop of bloom was collected with venipuncture, and the blood was stored in lysis buffer during transportation. The birds were treated with styptic powder and we ensured blood clotting before releasing each individual. All the blood sampling and processing were taken within 10 minutes at the natural breeding territory of each bird.
Field-collected samples	We extracted DNA from the blood samples (from each bird) that were kept in lysis buffer in the lab.
Ethics oversight	This research was conducted under approval of the UBC Animal Care Committee (protocol #A17-0049).

Note that full information on the approval of the study protocol must also be provided in the manuscript.
