# Supplementary material for: Signatures of mitonuclear coevolution in a warbler species complex
Source: Nat Commun. 2021 Jul 13;12:4279. doi: 10.1038/s41467-021-24586-8 (PMC8277850; doi:10.1038/s41467-021-24586-8)
Supplement: Supplementary file 4 — Description of Additional Supplementary Files [file 41467_2021_24586_MOESM4_ESM.pdf]

Description of Additional Supplementary Files

File Name: Supplementary Data 1
Description: Names and functions of genes involved in the 1.2 Mb chromosome 5 island of differentiation between inland STOW and coastal STOWs, as well as between inland STOW and SOCC. The Gene Ontology molecular and biological functions were based on *Taeniopygia guttata* homolog [2] from UniProt [3].

File Name: Supplementary Data 2
Description: Tab1: Accession numbers for *ND2* sequences used to evaluate evidence for positive selection with MEME. Tab2: Accession numbers for *ATP6* sequences used to evaluate evidence for positive selection with MEME.

File Name: Supplementary Data 3
Description: TreeSAAP analyses of physicochemical property of *ND2* and *ATP6*.

1. Wang, T., Hamann, A., Spittlehouse, D. L. & Murdock, T. Q. ClimateWNA-high-resolution spatial climate data for western North America. *J. Appl. Meteorol. Climatol.* **51**, 16–29 (2012).
2. Johnson, M. *et al.* NCBI BLAST: a better web interface. *Nucleic Acids Res.* **36**, W5–W9 (2008).
3. Bateman, A. UniProt: A worldwide hub of protein knowledge. *Nucleic Acids Res.* **47**, D506-515 (2019).
